# Antioxidant Potential and the Characterization of *Arachis hypogaea* Roots

**DOI:** 10.1155/2019/7073456

**Published:** 2019-12-20

**Authors:** Samee Ullah, Syed Ammar Hussain, Faryal Shaukat, Ahsan Hameed, Wu Yang, Yuanda Song

**Affiliations:** ^1^Colin Ratledge Center for Microbial Lipids, Center for Functional Foods and Health, School of Agriculture Engineering and Food Science, Shandong University of Technology, Zibo 255049, China; ^2^University Institute of Diet and Nutritional Sciences, Faculty of Allied Health Sciences, The University of Lahore, Lahore 54000, Pakistan; ^3^Clinical Research Center, Medical University of Bialystok, Bialystok 15-001, Poland

## Abstract

*Arachis hypogaea* roots are used as traditional Chinese medicine to treat different ailments, and the present study involves the exploration and comparison of phenolic profile and antioxidant activities (ABTS^+^ and DPPH assay) of *A. hypogaea* root extract in different solvents. 70% aqueous acetone and 70% aqueous ethanol were proved to be the best solvents to recover total phenolic compounds, with a yield of 42.59 ± 1.96 and 41.34 ± 0.92 mg/g dry weight of extract, respectively. ABTS^+^ radical scavenging activity was the highest in 70% aqueous ethanol, while the absolute methanol extract showed the highest DPPH radical scavenging activity (29.50 ± 2.19 *μ*g/mL). Furthermore, phytochemical profiling of 70% acetone extract of *A. hypogaea* roots was performed by LC-ESI-TOF-MS analysis which in turn indicated the presence of diverse compounds in the *A. hypogaea* root extract, namely, quinones, stilbenoids, and flavones and flavonoid glucosides.

## 1. Introduction

Food from plants, especially of medicinal plant origin, provides numerous biologically and naturally active compounds which promote health due to the presence of antioxidants, antihypertensive, sedative, antibacterial, anti-inflammatory, anticancer agents, etc. [1]. Phenolic compounds among other bioactive phytochemicals gained much attention recently due to their positive health effects including anti-inflammatory, antimicrobial, and antioxidant properties and their preventive role in metabolic syndromes [2]. And these compounds and their derivatives from plants were studied extensively to fulfill the need of natural antioxidant for pharmaceutical, cosmetic, food, and other applications [1]. Phenolic compounds and their metabolites, terpenoids, and alkaloids are three major classes of phytochemicals. These compounds are a source of antioxidants for humans as well as they protect vegetation from environmental stress [3]. Just like phenolic compounds, flavonoids also have several beneficial health effects such as anticancer and antiobesity effects and are beneficial against nicotine-related diseases [4]. Generous intakes of flavonoids are correlated with health benefits such as a lower risk of chronic conditions such as cardiovascular disease, stroke, and some cancers [5].

The ability to donate electron or hydrogen atoms or chelation of metal cations of any polyphenol represents its antioxidant capabilities [6]. The solubility of phenolic elements differs in different solvents due to the occurrence in different structures in different food and herb matrices. Hence, yield of polyphenols depends on the parameters like solvent polarity and isolation procedure. Soxhlet extraction, ultrasound-assisted extraction, supercritical fluid extraction, maceration, etc. are among the modern and conventional techniques used for the extraction of intracellular and extracellular antioxidant components from the plant. The variation in the yield is due to the complex nature of phytochemicals which may or may not solubilize in a given particular solvent, and ultimately, optimal extraction procedure differs for different herbs/plants [7, 8]. To recover the antioxidants from plant sources, mostly polar solvents are employed. The most suitable solvents for the maximum recovery of polyphenols are aqueous acetone, aqueous ethanol, aqueous ethyl acetate, and aqueous methanol. Ethanol is considered as good as well as safe, while methanol has been considered as an efficient way for extracting polyphenols with lower molar weight, and the acetone-water mixture is considered good for recovering antioxidants and flavonols with high molar weight [9]. The maximum recovery in terms of yield and total phenolic content from barley flour and legumes were obtained by ethanolic acetone and aqueous acetone, respectively [10].

*Arachis hypogaea* (i.e., groundnut) belongs to the legume family. The plant is rich in fibrous matter, albumin, starch, oil, and ash: soda, lime, magnesia, silica, phosphoric acid, chlorine, and sulphuric acid [11]. In traditional Chinese medicine, parts of *A. hypogaea* including roots are being used to treat different ailments. i.e., prostate enlargement, inflammation, and insomnia [12]. But it is rarely explored for its antioxidant and medicinal properties. In this study, the roots of *A. hypogaea* were extracted with different solvents to explore in vitro phenolic profile and antioxidant potential. Furthermore, LC-QTOF-MS analysis was also performed for the identification of different compounds present in the extract.

## 2. Materials and Methods

### 2.1. Materials and Reagents

*A. hypogaea* roots were provided by the local farmer from Shandong Province, China, and identification was authenticated by Haifang Xiao from SDUT. The chemicals used were of analytical grade or higher. Folin-Ciocalteu reagent, gallic acid, sodium carbonate (anhydrous), 2,2′-azinobis(3-ethylbenzothiazoline-6-sulfonic acid) or ABTS, anhydrous aluminum chloride, sodium nitrite (NaNO_2_), calcium carbonate, copper (II), (+)-catechin, vanillin, ascorbic acid, HCl, NaOH, quercetin, trolox, DPPH (2,2-diphenyl-1-picrylhydrazyl), potassium persulphate, and potassium phosphate were purchased from Sigma-Aldrich (Steinheim, Germany). Formic acid was purchased from Merck (Darmstadt, Germany), and HPLC-grade acetone, ethanol, methanol, and acetonitrile were supplied by Sigma-Aldrich (Steinheim, Germany).

### 2.2. Sample Preparation and Extraction

Fresh *A. hypogaea* root samples were sorted, washed, and shade-dried at room temperature for 12 days to remove the contaminants. The dried *A. hypogaea* roots were then grounded to obtain the crude powder. The crude powder was stored at −20°C until used further for extraction. 2 g freshly grounded samples was extracted with 20 ml of different solvent systems (see Table 1, for detail) in dark, at room temperature under constant agitation for 18–24 hours. The extracts were centrifuged at 6500 rpm for 25 minutes, the procedure was repeated twice, and supernatants were combined. Residues from the above step were again subjected to extraction by the addition of another 20 mL of solvent, and supernatants were combined with the previous extracts. In a preweighed glass plate, 20 mL of the pooled extracts was then taken and solvents were evaporated and dried at 35 ± 5°C until complete dryness. The difference in mass after drying was used to calculate the total extractable mass.

### 2.3. Estimation of Antioxidant Components

#### 2.3.1. Total Phenolic Contents

Total phenolic compounds were determined by the Folin-Ciocalteau method previously described by Singleton et al. [13] with minor modifications. Briefly, 3 mL of Folin-Ciocalteau reagent (1 : 10 in water) was mixed with 1 mL sample extracts. After the incubation at room temperature for 5 minutes, the mixture was then placed in the dark for one hour before taking the final absorbance at *λ* 765 nm. Finally, absorbance values were compared with the standard curve of gallic acid prepared in the extraction solvent over the range of 1–100 *μ*g/mL. The total phenolic contents (TPC) in the extract were expressed in equivalence to gallic acid (mg gallic acid/g dry weight of extract).

#### 2.3.2. Total Flavonoid Contents

The AlCl_3_ method reported by Quettier-Deleu et al. [14] was used to express the total flavonoid contents (TFC). Briefly, the ethanolic solution of AlCl_3_·6H_2_O (2% v/v) was mixed with 0.5 mL of each extract (10 mg/mL), and absorbance was taken at *λ* 430 nm, after 10 min of incubation at room temperature. Total flavonoids were calculated and expressed in quercetin equivalent, by comparing with the quercetin (10–50 *μ*g/mL) standard curve.

#### 2.3.3. Total Condensed Tannins

After dissolving each dried extract in its respective solvent, a 4% vanillin solution (1.5 mL) was mixed with 50 *μ*L of each extract, followed by the mixing of concentrated HCl (0.75 mL). The previously described homogeneous mixture was incubated for 20 min in darkness, at room temperature. A standard curve was made with 50–500 *μ*g/mL catechin, while a 4% (v/v) solution of concentrated HCl in ethyl alcohol was used as blank. The absorbance was taken at *λ* 500 nm against blank, and the total condensed tannins (TCT) were expressed in equivalence to catechin as mg/g dry weight of extracts [15].

### 2.4. Antioxidant Assays

#### 2.4.1. ABTS^+^ Radical Scavenging Activity

ABTS^+^ radical scavenging assay was performed according to our previous investigation [16]. A stock solution of 7 mM concentration of ABTS^+^ (2,20-azinobis) was prepared by dissolving ABTS in water. ABTS and 2.45 mM K_2_S_2_O_8_ in the ratio of 1 : 1 (v/v) were thoroughly mixed to produce ABTS^+^, 12–16 hours prior to performing the analysis. To reach the absorbance 0.7 ± 0.2 at *λ* 734 nm, dilution of previously made ABTS^+^ was done with 95% ethanol. A ratio of 9 : 1 ABTS^+^ reagent to extract was used to perform the analysis. And the results were expressed in *μ*g/mL concentration of phenolic compounds which was effective to inhibit half (IC_50_) of ABTS^+^ radicals. The following equation was used to calculate ABTS^+^ radical scavenging activity:

(1) $$\begin{array}{c} E\;=\;\frac{\left(A_{C}-A_{s}\right)}{A_{o}}\;*\;100, \end{array}$$

where *A*_c_ = absorbance of trolox, *A*_0_ = absorbance of the negative control, and *A*_s_ = sample's absorbance.

#### 2.4.2. DPPH Free Radical Scavenging Activity

For the estimation of DPPH antioxidant activity, Shimada et al.'s [17] method was used with minor alterations. Briefly, 100 mL methanol was used to dissolve 12 mg DPPH to get the 0.3 mM concentration of DPPH. The extracts (each 2.5 mL; 20–140 *μ*g/mL) were mixed with 2.5 mL DPPH solution in a glass tube and incubated for 20 min in darkness prior to measuring absorbance. Absorbance was measured at *λ* 517 nm to calculate the decrease in absorbance after incubation. DPPH activity was expressed as *μ*g/mL of phenolic compounds which effectively inhibit the 50% of free radicals. Percent inhibition was calculated as follows:

(2) $$\begin{array}{c} \text{\% inhibition}=\frac{\left(A_{c}-A_{s}\right)}{A_{c}}\;\times \;100, \end{array}$$

where *A*_C_ = absorbance of DPPH solution and *A*_S_ = absorbance of the sample with DPPH solution.

### 2.5. LC–ESI-QTOF-MS Analysis

For LC-ESI-QTOF-MS analysis, 70% aqueous acetone extract was used for the profiling of extract using an Agilent 1100 Liquid Chromatography system (Agilent Technologies, Palo Alto, CA, USA) furnished with a standard auto-sampler as described in our previous study [16] with some alterations. Briefly, the analytical column used was characterized as Phenomenex Gemini C18 (1.7 *μ*m, 2 × 100 mm) operated at 45°C with a gradient elution portfolio at a flow rate of 0.5 mL/min. The mobile phases used were water and 0.1% formic acid (A) and acetonitrile and 0.1% formic acid (B). A 10 *μ*L sample volume injection was used, and the flow rate was 0.5 mL/min. Multistep gradient was applied in the following fashion: at time 0 min, 10% B; 5 min, 20% B; 10 min, 35% B; 21 min, 45% B; 24 min, 55% B; 28 min, 75% B; and 32 min, 90% B. The final condition was maintained for the next 8 minutes [18].

### 2.6. Statistical Analysis

All experiments were conducted in triplicate, and one-way analysis of variance using Tukey's multiple comparison tests was performed. Results were presented as mean ± SD. GraphPad Prism 6 (GraphPad Software, San Diego, CA, USA, http://www.graphpad.com) was used for all the calculations. Significance was observed at *p* < 0.05.

## 3. Results and Discussion

### 3.1. Yield

**Figure 1:**
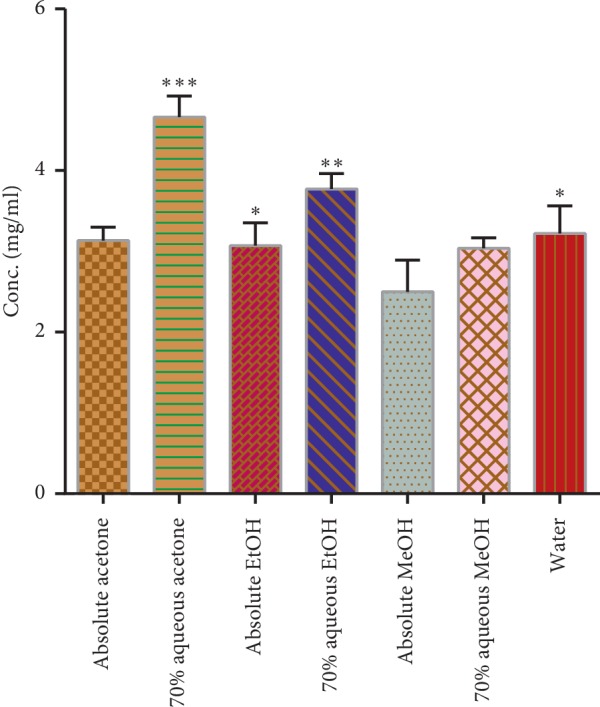
Yield of *A. hypogaea* extracts in mg/mL. Asterisks indicate the difference is significant among the means of yield (^*∗*^*p* < 0.005; ^*∗∗*^*p* < 0.001; ^*∗∗∗*^*p* < 0.0001).

There are numerous steps to obtain the phytochemicals from a plant such as milling, grinding, homogenization, and extraction. Among them, extraction is the first major step for recovering and isolating the phytochemicals from plant materials. The efficiency of extraction procedure depends on the chemical nature of the phytochemicals, method of extraction employed, the size of sample particles, the solvent employed, and other interfering substances present in the food/plant material [19]. The extraction yield also depends on other factors such as solvent polarity, pH value, temperature, and extraction time. Under the same extraction time and temperature, the solvent and composition of the sample are known as the most important parameters. These aforesaid factors not only influence the extraction yield but also the functionality and efficiency of resulting extracts. There are a lot of techniques available, both conventional and advanced, in the literature. We had selected a conventional extraction technique for our studies as described by Hameed et al. and Hussain et al. [16, 20]. The adaptation of these techniques with minor modifications provided us with significantly different yields with different solvents. The extraction yield in different solvent systems ranged from lowest 2.5 ± 0.39 mg/mL in methanol to highest 4.66 ± 0.17 mg/mL in 70% aqueous acetone [21]. The yield of the *A. hypogaea* root extracts increased in different solvent systems in the following manner: methanol <70% methanol < acetone < ethanol < water <70% ethanol <70% acetone. There was a significant variation between the yields of different solvent extracts (Figure 1).

### 3.2. Total Phenolic Contents

**Figure 2:**
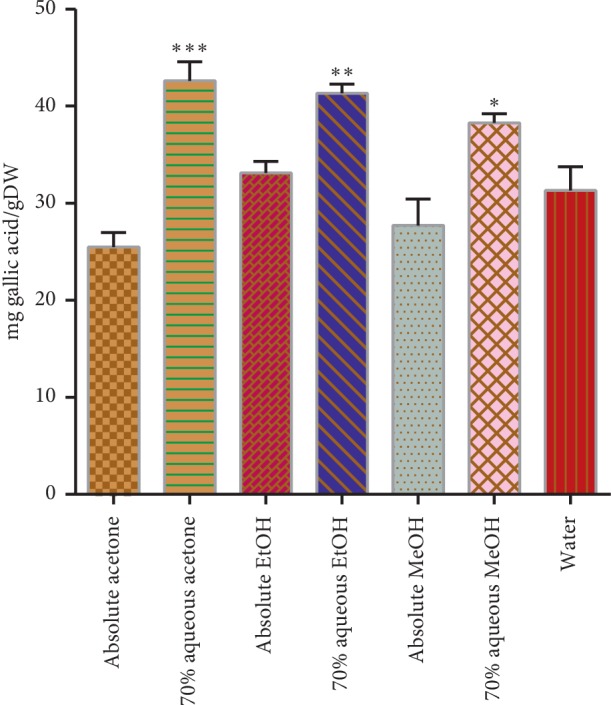
Total phenolic contents in mg gallic acid/g dry weight of the extract. Asterisks indicate the difference is significant among the means of TPC (^*∗*^*p* < 0.005; ^*∗∗*^*p* < 0.001; ^*∗∗∗*^*p* < 0.0001).

In the current study for the extraction of the phenolic compounds from the *A. hypogaea* roots, different solvent systems were used under the same conditions. Total phenolic contents of the extracts were significantly different (*p* < 0.05) with the composition of solvents, and they were ranged from the lowest (25.46 ± 1.5) to highest (42.59 ± 1.96 mg/g) dry extract (Figure 2). The best solvent was 70% aqueous acetone for the extraction of phenolic compounds in this current investigation. It is well known that solvent polarity will play a key role in increasing phenolic solubility and acetone-water mixtures which were very effective solvents in our study and also reported as a good solvent for the extraction of polar antioxidants from the fruits, vegetables, and legumes [22]. However, in another study, it was reported that the best solvents for phenolic extraction from horseradish roots were ethanol and ethanol/water solutions [23]. In other studies conducted by some groups, acetone-based mixtures were also found to be more effective solvents than the methanol-based mixtures for phenolic extraction from fruits and vegetables [24]. Additionally, the maximum polyphenolic extraction yield was obtained in the methanol extract of bauhinia vahlii followed by acetone, hot water, and chloroform extracts [25]. The total phenolic contents are higher in 70% aqueous acetone extract of *A*. *hypogaea* roots as compared to the total phenolic contents reported in *Withania somnifera* [26].

### 3.3. Total Flavonoid Contents

**Figure 3:**
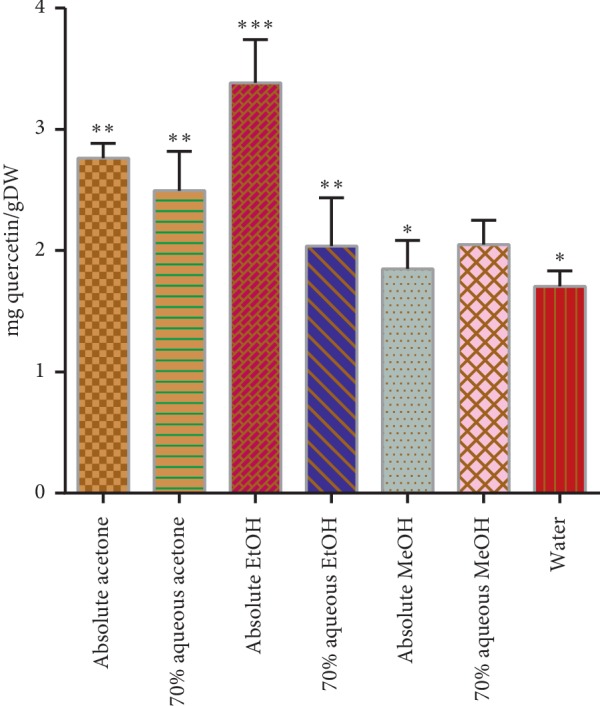
Total flavonoid contents in mg quercetin/g dry weight of the extract. Asterisks indicate the difference is significant among the means of TFC (^*∗*^*p* < 0.005; ^*∗∗*^*p* < 0.001; ^*∗∗∗*^*p* < 0.0001).

The total flavonoid contents (TFC) from the *A. hypogaea* root extracts are presented in Figure 3. The highest level of total flavonoid contents was present in absolute ethanol (3.38 ± 0.35 mg quercetin/g dry weight) which was significantly (*p* < 0.05) different from the other solvent systems, while the lowest was found in water (1.70 ± 0.13 mg quercetin/g dry weight). These findings are supported by the previously reported investigations [27]. Except 70% aqueous methanol, the TFC decreased with the increasing water contents in the solvents, while with the increase of water content in methanol, the TFC also increased.

### 3.4. Total Condensed Tannins

After lignin, cellulose, and hemicellulose, the most abundant component in biomass is tannin. And after lignin, they represent the second more extensive source of phenolic components [28]. In the aquatic and terrestrial environment of the vegetable kingdom, tannin is well distributed. Tannins have phenolic features due to the presence of the phenolic rings in their structure, which is responsible for scavenging the electrons or radical ions. Medical, pharmaceutical, and food industries widely use tannins due to its antioxidant nature [29].

**Figure 4:**
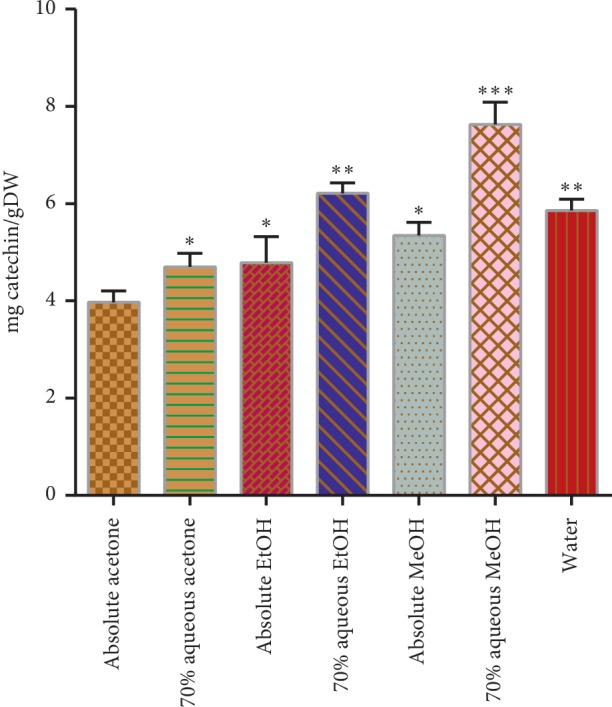
Total condensed tannins in mg catechin/g dry weight of the extract. Asterisks indicate the difference is significant among the means of TCT (^*∗*^*p* < 0.005; ^*∗∗*^*p* < 0.001; ^*∗∗∗*^*p* < 0.0001).

The condensed tannin contents (TCT) of the present study are presented in Figure 4. The best yield for condensed tannin as catechin equivalent was obtained from 70% aqueous methanol (7.63 ± 0.45 mg/gDW) which is significantly different (*p* < 0.05) from the other solvent systems, followed by the 70% aqueous ethanol (6.21 ± 0.21 mg/gDW), while the least condensed tannins were observed in absolute acetone extract, i.e., 3.97 ± 0.22 mg/gDW. The aforesaid trend was previously reported by de hyoyos-Martinez et al. [30]. The tannin content increases with the increasing polarity of the solvent.

### 3.5. Antioxidant Activity

#### 3.5.1. ABTS^+^ Free Radical Scavenging Activity

**Figure 5:**
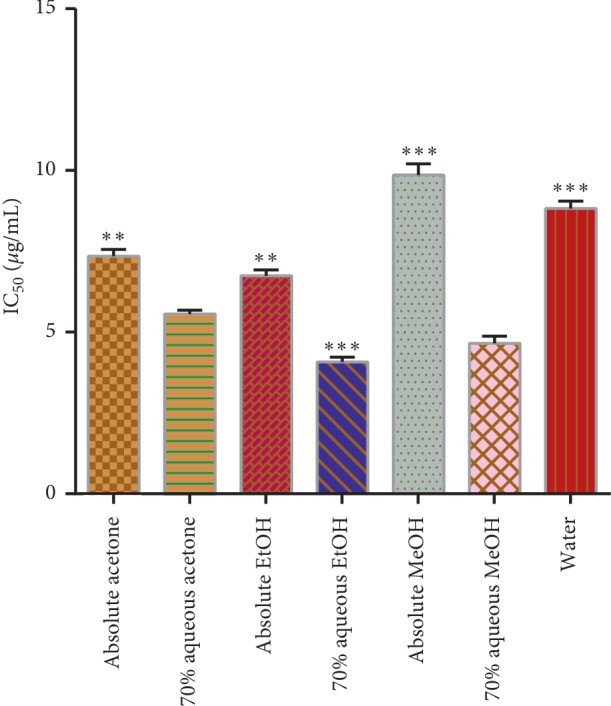
ABTS^+^ radical scavenging activity in *μ*g/mL of the extract. Asterisks indicate the difference is significant among the means of effective concentration to inhibit half of ABTS^+^ free radical scavenging activity (^*∗∗*^*p* < 0.001; ^*∗∗∗*^*p* < 0.0001).

The ABTS^+^ assay measures the relative ability of antioxidants to scavenge ABTS^+^ generated in the aqueous phase, as compared with a trolox (water-soluble vitamin E analog) standard. The ABTS^+^ is generated by reacting with a strong oxidizing agent (e.g., potassium permanganate or potassium persulphate) with the ABTS salt [31]. The concentrations used to inhibit half of the free radicals (ABTS^+^) are expressed in Figure 5 as IC_50_. The best inhibitory effect was shown by the 70% aqueous ethanol extract in low concentration (4.08 ± 0.16 *μ*g/mL) followed by the 70% aqueous methanol extract (4.66 ± 0.22 *μ*g/mL). A significant difference in inhibitory concentrations was recorded (*p* < 0.05) among the different solvent extracts.

#### 3.5.2. Scavenging Activity of DPPH^+^

Stable organic DPPH free radical absorbs light at 517 nm. Noticeable discoloration in the color (purple to yellow) of DPPH can be observed when it loses the electron and ultimately loses its absorption at 517 nm. To detect the ingredients at low concentration, it is sensitive enough to do so [32].

**Figure 6:**
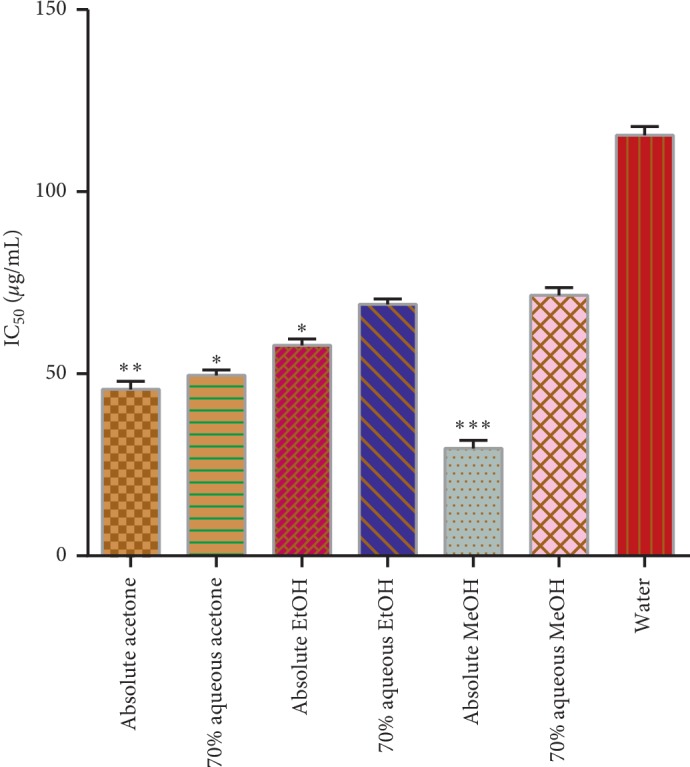
DPPH radical scavenging activity in *μ*g/mL and percent inhabitation. Asterisks indicate the difference is significant among the means of effective concentration to inhibit 50% of free radicals (^*∗*^*p* < 0.005; ^*∗∗*^*p* < 0.001; ^*∗∗∗*^*p* < 0.0001).

Figure 6 depicts the scavenging activity of DPPH. The concentration of the extracts which provided 50% inhibition (IC_50_) is inversely proportional to the DPPH activity; the higher the concentration of the extract used, the lower the DPPH scavenging activity will be. In our study, the absolute methanol extract showed the highest DPPH scavenging activity in terms of effective dose (EC_50_) which inhibits 50% of the free radicals (IC_50_) with the minimum extract (29.50 ± 2.19 *μ*g/mL); a significant difference was observed among the different solvent extracts at *p* < 0.05 while the lowest inhibition was shown by water extract and the same IC_50_ was recorded with much higher extract concentration, i.e., 116.21 ± 2.84 *μ*g/mL. Alothman et al. [33] and some other researchers also reported similar trends while working on DPPH scavenging activity of pineapple crude extracts and wheat germs. This in turn supported the findings of the current investigation.

### 3.6. Compounds Profiled by LC-QTOF-MS Analysis

**Figure 7:**
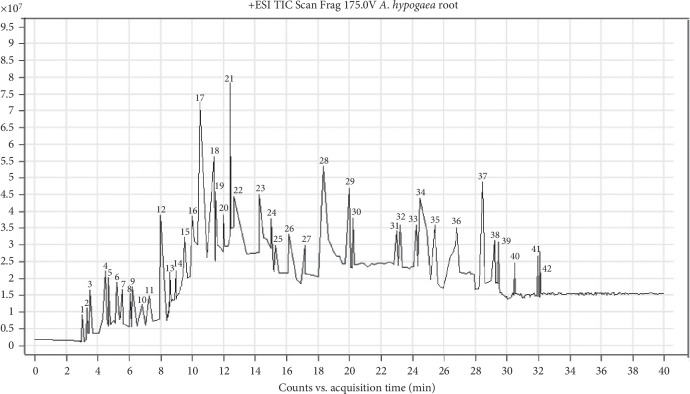
Chromatogram from LC-QTOF-MS analysis.

The 70% aqueous acetone extract of *A. hypogaea* root is subjected to LC-QTOF-MS analysis. The chromatogram turned up by the LC-QTOF-MS analysis is shown in Figure 7. By comparing the mass spectra and retention times with the literature data, reference standards, and database, peaks were identified for reference compounds. Forty-two compounds were identified from the 70% aqueous acetone macerated extract (Table 2) by using Agilent Technology's mass hunter software. Peak numbers, compositions, compound names, their retention time, and experimental m/z values along with difference/error are also presented in Table 2. Diverse classes including alkaloids, terpenes, quinone, coumarins and lignans, flavone and flavonoid glucosides, lipids, stilbenoids, and other miscellaneous compounds were identified from the macerated acetone extract.

Peak 1 (compound: 3,4-dihydroxy-acetophenone, m/z: 162.1724) is a glucoside phenolic compound present in coffee, coffee products, and picea pungen's needles and was reported to improve the endothelial function in diabetes-induced rats [34]. Peak 2 (compound: dihydrocaffeic Acid, m/z: 182.13) is an organic phenolic and metabolite of caffeic acid, belongs to catechol, is present in plants because in lignin synthesis, caffeic acid is the intermediate product, has been previously reported for its antioxidant potential at high temperature, and is a xenobiotic metabolite in humans [35].

Peak numbers 3, 9, 12, 15, 18, 20, 23, 24, 25, 31 and 36 represent different flavonoids and their derivatives with numerous health beneficial properties including antioxidant, anticancer, anti-inflammatory, antidiabetic and antimicrobial properties [36]. 2-Methoxy cinnamic acid (peak 5, m/z: 178.195) is a bioactive derivative of cinnamic acid and metabolite of tyrosinase inhibitor and had shown anti-inflammatory, antipyretic, and analgesic properties [37]. Compounds panaxytriol (peak 14, m/z: 277.8031) and fraxinellonone (peak 16, m/z: 179.9755) have antiproliferative and anti-inflammatory and insecticide potential [38]. Peak numbers 17 and 21 were dihydroresveratrol and resveratrol with m/z: 246.6983 and 228.0796, respectively; they are the nonflavonoid polyphenols and stilbenoids and were previously reported in grapes, wine, berries, and *A. hypogaea*. The extensive study showed their potential as antioxidant, anticarcinogenic (reduce the risk of prostate cancer) and anti-inflammatory, and anti-alzheimer and cardiovascular diseases [39].

Peak numbers 22, 28, 40, 41, and 42 represented the presence of different fatty acids in the *A. hypogaea* root extract; among them, some have health benefits including anti-inflammatory and anti-carcinogenic properties [40, 41]. Moreover, arnebinone (peak 29, m/z: 302.9837) and triterpenoid lupenone (peak 37, m/z: 424.8617) are reported to have anti-inflammatory, antiedema, antiarthritic, and antitussive activities, and studies also showed that it can be used to treat the hypopigmentation disease because lupenone stimulates the melanogenesis [42, 43].

Cerevisterol (peak 39, m/z: 430.697) was originally described and isolated from fungus (Xylaria species) in the 1930s and considered as therapeutic agent against cancer and tumor, but the presence in the *A. hypogaea* roots extract is the most probably due to fungal growth on the plant roots and ultimately incorporation of the aforesaid compound occurred in the extract [44]. Due to the heterogeneous occurrence of substances inside the plants, some compounds were identified more than once in the form of different metabolites.

## 4. Conclusion

Our study on the root extract of *A. hypogaea* clearly indicates that *A. hypogaea* roots are a rich source of antioxidants. Almost all the extracts showed reasonably high TPC, TFC, TCT, ABTS, and DPPH activities. The highest yield and total phenolic contents were recorded in 70% aqueous acetone extract. Both solvents, the 70% aqueous acetone and 70% aqueous ethanol, were found to be good solvents for the extraction of antioxidant compounds from *A. hypogaea* roots. From LC-QTOF-MS analysis, numerous compounds with great biological activities, i.e., anticancerous, anti-inflammatory, and sedative agents, were identified in the extract of *A. hypogaea* root extract. Use of *A. hypogaea* roots as traditional Chinese medicine for health benefits is justifiable and in the future, it can be used in the development of nutraceutical products after quantitative exploration of the compounds.

## Figures and Tables

**Table 1:** Composition of solvents used for extraction.

Solvents	Composition
1	Absolute acetone
2	70% aqueous acetone
3	Absolute ethanol
4	70% aqueous ethanol
5	Absolute methanol
6	70% aqueous methanol
7	Water

**Table 2:** Compound identified in LC-ESI-QTOF-MS/MS analysis.

Peak number	Composition	Compound	Retention time (min)	M/Z [M+H]^+^	Difference
1	C_10_H_10_O_2_	2,4-Dihydroxyacetophenone	2.98	162.1724	0.10
2	C_9_H_10_O_4_	Dihydrocaffeic acid	3.33	182.13	0.073
3	C_15_H_10_O_6_	Kaempferol	3.49	286.0158	−0.03
4	C_21_H_22_O_9_	Aloin	4.48	418.2369	0.12
5	C_10_H_10_O_3_	2-Methoxy cinnamic acid	4.67	178.195	0.13
6	C_23_H_33_NO	Evocaprine	5.18	339.4653	0.21
7	C_13_H_10_O	Atractylodin	5.57	182.2184	0.15
8	C_22_H_33_O_11_	Peonidin-3-o-glucoside	6.06	463.2339	0.11
9	C_15_H_10_O_5_	4′,5,7-Trihydroxyflavone	6.18	270.932	0.88
10	C_16_H_12_O_5_	Questin	6.79	284.0767	0.008
11	C_29_H_37_N_3_O_3_	Tubulosine	7.28	475.4939	0.21
12	C_17_H_16_0_6_	Persicogenin	7.98	315.9347	−0.16
13	C_13_H_18_N_2_O_4_	Indolol	8.57	266.6059	0.31
14	C_17_H_25_O_3_	Panaxytriol	8.95	277.8031	0.62
15	C_16_H_14_O_5_	Isosakuranetin	9.51	286.7993	0.71
16	C_11_H_16_O_2_	Fraxinellonone	10.01	179.9755	−0.13
17	C_14_H_14_O_4_	Dihydroresveratrol	10.48	246.6983	0.60
18	C_17_H_16_H_5_	5-Hydroxy-4′,7-dimethoxyflavanone	11.38	300.0194	−0.078
19	C_15_H_22_0_10_	Catalpol	11.48	362.3748	0.25
20	C_25_H_26_O_5_	Cajaflavanone	11.96	406.7547	0.58
21	C_14_H_12_O_3_	Resveratrol	12.43	228.0796	0.002
22	C_16_H_28_O_3_	13-Hydroxy-9,11-hexadecadienoic acid	12.64	268.7196	0.5
23	C_16_H_14_O_5_	Sakuranetin	14.25	286.2835	0.19
24	C_15_H_10_O_6_	Scutellarin	15.03	285.1583	−0.8
25	C_15_H_10_O_6_	Tetra hydroxyl flavone	15.30	287.7189	1.67
26	C_14_H_20_O_7_	Salidroside	16.13	300.0465	−0.07
27	C_18_H_28_0_2_	Ambrettolide	17.15	277.3101	1.10
28	C_18_H_30_O_2_	Punicic acid	18.33	278.8491	0.63
29	C_18_H_22_O_4_	Arnebinone	19.98	302.9837	0.84
30	C_20_H_26_O_3_	Oxyphyllacinol	20.22	314.6153	0.42
31	C_20_H_18_O_6_	Luteone	22.98	354.188	0.08
32	C_24_H_26_O_7_	Anomalin	23.20	426.7432	0.58
33	C_22_H_22_O_7_	Anthricin	24.24	397.1134	−1.02
34	C_17_H_24_O_3_	Shogaol	24.48	277.278	1.1
35	C_8_H_8_O_3_	4-Methoxybenzoic acid	25.39	152.2951	0.25
36	C_34_H_30_O_14_	3-(2,3-Diacetyl-4-P-coumaroylrhamnoside)	26.82	662.4490	−0.43
37	C_30_H_48_O	Lupenone	28.45	424.8617	0.49
38	C_16_H_18_O_10_	Fraxin	29.21	370.1531	0.06
39	C_28_H_46_O_3_	Cerevisterol, Α-sitosterol	29.43	430.697	0.36
40	C_18_H_32_O_2_	Linoleic acid	30.48	280.3564	0.11
41	C_16_H_32_O_2_	Palmitic acid	31.95	256.1528	−0.08
42	C_18_H_34_O_2_	Oleic acid	32.11	281.1362	−0.12

## Data Availability

The data that support these findings have been provided in the supplementary materials.

## Supplementary Materials

Supplementary Materials Table 1: extraction yield, antioxidants, and antioxidant activities of *A. hypogaea* in different solvent systems.
