# Supplementary material for: Antioxidant Potential and the Characterization of Arachis hypogaea Roots
Source: Biomed Res Int. 2019 Dec 20;2019:7073456. doi: 10.1155/2019/7073456 (PMC6948283; doi:10.1155/2019/7073456)
Supplement: Supplementary Materials — Table 1: extraction yield, antioxidants, and antioxidant activities of A. hypogaea in different solvent systems. [file 7073456.f1.pdf]

Supplementary Table

Table1. Extraction yield, antioxidants and antioxidant activities of A. hypogaea in different solvent systems

Solvent	[1]Yield	[2]TPC	[3]TFC	[4]TCT	[5]ABTS$^+$	[6]DPPH
**Absolute Acetone**	3.13±0.17^{c}	25.46±1.5^{d}	2.76±0.12^{a}	3.97±0.22^{a}	7.35± 0.21^{a}	45.71±2.21^{a}
**70% aqueous Ace**	4.66± 0.26^{a}	42.59±1.96^{a}	2.49±0.32^{ab}	4.69±0.28^{ac}	5.54±0.12^{b}	49.64±1.44^{a}
**Absolute EtOH**	3.06 ± 0.28^{c}	33.10±1.19^{c}	3.38±0.35^{a}	5.51±0.37^{ac}	6.74±0.19^{a}	57.78±1.92^{b}
**70% aqueous EtOH**	3.77± 0.23^{b}	41.34± 0.92^{a}	2.03±0.4^{b}	6.21±0.21^{bc}	4.08±0.16^{c}	69.06±1.48^{c}
**Absolute MeOH**	2.51±0.39^{d}	27.95± .78^{d}	1.85±0.24^{b}	5.34±0.27^{c}	9.86±0.35^{d}	29.50±2.19^{d}
**70% aqueous MeOH**	3.04± 0.12^{c,d}	38.26± 0.95^{b}	2.11±0.19^{b}	7.63±0.45^{d}	4.63±0.25^{c}	71.56±2.08^{ce}
**Water**	3.22±0.35^{c}	29.31±1.45^{d}	1.70±0.13^{c}	5.85±0.23^{bc}	8.82±0.23^{e}	116.21±2.84^{f}

Note: All the results are expressed as mean ± S.D. (n= 3). [1]Yield expressed as mg/mL, [2]TPC= total phenolic contents expressed in mg gallic acid/ gram dry extract, [3]TFC= Total flavonoid contents expressed in mg quercetin/ gram dry weight of extract, [4]TCT= total condensed tannins expressed in mg Catechin/ gram dry extract. [5]ABTS$^+$ and [6]DPPH expressed as effective concentration (µg/mL) to inhibit 50% of free radicals. The one-way analysis of the variance followed by Tuckey's multiple comparison test was performed to evaluate significance at $p<0.05$, different superscripted letters within the same column indicate significant difference.
